# Percutaneous stereotactic image-guided microwave ablation for malignant liver lesions

**DOI:** 10.1038/s41598-019-50159-3

**Published:** 2019-09-25

**Authors:** Stéphanie Perrodin, Anja Lachenmayer, Martin Maurer, Corina Kim-Fuchs, Daniel Candinas, Vanessa Banz

**Affiliations:** 1 Department of Visceral Surgery and Medicine, Inselspital, Bern University Hospital and University of Bern, Bern, Switzerland; 2 Department of Radiology, Inselspital, Bern University Hospital and University of Bern, Bern, Switzerland

**Keywords:** Targeted therapies, Surgical oncology

## Abstract

Thermal ablation has proven beneficial for hepatocellular carcinoma and possibly for colorectal liver metastases, but data is lacking for other liver metastases. Computer-assisted navigation can increase ablation efficacy and broaden its indications. We present our experience with percutaneous stereotactic image-guided microwave ablation (SMWA) for non-colorectal liver metastases (NCRLM), in form of a retrospective study including all SMWA for NCRLM from 2015 to 2017. Indication for SMWA was determined at a multidisciplinary tumorboard. End-points include recurrence, overall and liver-specific disease progression and complications. Twenty-three patients underwent 25 interventions for 40 lesions, including 17 neuroendocrine tumor, nine breast cancer, four sarcoma, two non-small cell lung cancer, three duodenal adenocarcinoma, one esophageal adenocarcinoma, one pancreatic adenocarcinoma, one ampullary carcinoma, one prostate carcinoma, and one renal cell carcinoma metastases. Median follow-up was 15 months (2–32). Incomplete ablation rate was 2.5% (1/40), local recurrence rate 10% (4/40). Three patients (12%) had minor complications. Overall disease progression was 73.9% (17/23), median disease-free survival 7 months (0–26) and overall survival 18 months (2–39). SIMWA is feasible, safe and minimally invasive for NCRLM in selected patients. While it might offer an alternative to resection or palliative strategies, the oncological benefit needs to be evaluated in a larger patient cohort.

## Introduction

Ablation is an increasingly recognized alternative to surgery for liver tumors in patients with impaired liver function, associated extrahepatic disease, lesions inaccessible to surgical resection, extensive bilobar metastatic disease or concurrent medical conditions precluding an operation^1^.

Thermal Ablation techniques include radiofrequency ablation (RFA) and microwave ablation (MWA). The advantages of MWA are the potential to treat larger tumors faster, with a reduced perivascular heat sink effect^2^. MWA is well recognized for hepatocellular carcinoma (HCC), where it has been shown to be a safe, effective and minimally-invasive treatment option, that can be repeated in case of local recurrence^3^. It is also increasingly recognized for colorectal liver metastases (CRLM), where it is being investigated in the setting of prospective randomized clinical trials, comparing surgery to thermal ablation, such as the COLLISION Trial^4–7^.

Furthermore, the use of MWA for the treatment of other liver metastases with curative intent, such as is the case for hepatic neuroendocrine tumor (NET) metastases, is gaining popularity. When feasible, treatment with curative intent for hepatic NET metastases consists in (if necessary, repeated) liver resection, resulting in improved survival^8–10^. However, while current data show that RFA of NET liver metastases may be beneficial, there is still no conclusive evidence on the efficacy of MWA for such lesions^11^. Data is even scarcer on the treatment of other hepatic tumor entities using MWA and the benefit of such local treatment strategies remains unproven.

More recently, image-guided ablation is increasingly being combined with computer-assisted stereotactic navigation^12^. Computer-assisted stereotactic navigation is particularly interesting in the setting of very small or invisible “vanishing” lesions (targeting accuracy), very large lesions requiring multiple needle placements in order to achieve complete ablation, or difficult-to-reach or treat lesions (close proximity to major vessels/bile ducts, liver dome, segment I lesions). The use of computer-assisted stereotactic navigation has shown to improve the precision of the needle placement^13–15^, but data is lacking on complete ablation rates and long-term results.

The aim of our study is to analyze our cohort of patients with liver metastasis of non-colorectal, non-HCC origin treated with stereotactic image-guided microwave ablation (SMWA) between 2015 and 2017.

## Methods

Between January 2015 and December 2017, 163 patients with 286 lesions were treated with SMWA in our institution, a certified cancer center. Most patients had HCC or CRLM. We performed a retrospective analysis of prospectively collected data from all patients undergoing SMWA for liver metastases of non-colorectal, non-HCC origin in this time period. SMWA was carried out as per recommendation by our multidisciplinary tumorboard. Obligatory participants include a radiologist, an interventional radiologist, a hepatologist, an oncologist, a radiooncologist, a pathologist and a hepatobiliary surgeon. Exclusion criteria were cross-sectional images (CT or MRI) indicative for, or biopsy proven HCC, CRLM or benign lesions. Clinical data was obtained from the electronic patient files.

End-points included local recurrence rate at the site of ablation, appearance of new intrahepatic or extrahepatic lesions, post-interventional complications according to the Clavien-Dindo Classification^16^ and hospital length of stay.

Follow-up consisted of 3-monthly clinical and radiological (CT scan or MRI) check-ups. Additional oncological follow-up was carried out at the discretion of and as recommended by the treating oncologist.

Incomplete ablation was defined as detectable tumor on the edge of the ablation zone on the first post-SMWA follow-up image. An initial imaging is carried out directly post-ablation while the patient is still in the CT suite, allowing for immediate validation of treatment efficacy. Local recurrence was defined as the presence of a detectable tumor within 10 mm from the edge of the ablation zone^17^.

The study protocol was approved by the Regional Ethical Review Boards in Bern (Kantonale Ethikkommission Bern, KEK-Nr. 2017-01038). General consent was obtained at time of hospitalisation, but specific consent was waived due to the retrospective study design. The study was performed in accordance with the relevant national guidelines and regulations.

### Stereotactic image-ablation

All CT-guided interventions were performed by a dedicated interdisciplinary team of surgeons and radiologists, based on CT imaging. Trajectory planning, probe positioning and validation of treatment was conducted using a commercially available navigation system for interventional radiology (CAS-ONE, CASination AG, Bern, Switzerland). Microwave energy (Acculis MTA System, AngioDynamics, Latham, NY, USA) was used.

Interventions were performed under general anesthesia, and patients were ventilated using high frequency jet ventilation, ensuring minimal diaphragmatic movement during the procedure.

The procedure included the following steps: Planning of the trajectory of the ablation needle, navigated positioning of the ablation needles in the lesion, validation of their localisation in the liver and ablation of the lesion using 100 W energy. The duration of ablation was determined according to the size of the lesion, the proximity to blood vessels or bile ducts and the quality of the surrounding liver tissue. Finally, the pre- and post-ablation CT scans were overlayed to validate the ablation zone.

### Statistical analysis

Descriptive statistics were used to present patient characteristics and results. Continuous data is presented as total number, percentage, mean and standard deviation or median and range. All analyses were performed using a commercially available software (SPSS version 25).

## Results

### Patient characteristics

In total, 23 patients required 25 interventions to treat 40 malignant liver lesions of non-colorectal, non-HCC origin.

Most patients were male, median age was 61 years and 74% of patients presented with significant comorbidities, ASA III or more (Table 1)^18^.

**Table 1:** Patients characteristics (n = 23).

Variable	
Mean age, years (range)	58.4 (7–79)
Male gender, n (%)	13 (56.5)
ASA classification, n (%)	
ASA II	6 (26.1)
ASA III	16 (69.6)
ASA IV	1 (4.3)
Identification of liver metastases, n (%)	
Before primary tumor	1 (4.3)
At the same time	2 (8.7)
After primary tumor	20 (87)
Median time between initial diagnosis and metastasis, months (range)	19 (0–312)
Previous treatment for liver metastases, n (%)	5 (21.7)
IRE	1 (4.3)
Surgical resection only	2 (8.7)
Surgical resection and open MWA	1 (4.3)
Laparoscopic MWA	1 (4.3)
Extra-hepatic disease at time of SMWA, n (%)	3 (13)
Systemic therapy at time of SMWA, n (%)	7 (30.4)

*ASA* American Society for Anesthesia, *IRE* irreversible electroporation, *MWA* Microwaveablation.

In 87% of patients the liver metastases were diagnosed 3 to 312 months after initial diagnosis of the primary tumor. In two patients the liver metastases and the primary tumor were diagnosed at the same time. In one patient the liver metastasis was diagnosed prior to identification of the primary tumor. Five patients underwent previous local treatment for liver metastases, such as laparoscopic MWA or surgical resection. Three patients had stable extrahepatic disease at the time of SMWA, and almost one third of patients underwent SMWA while under systemic therapy.

**Figure 1:**
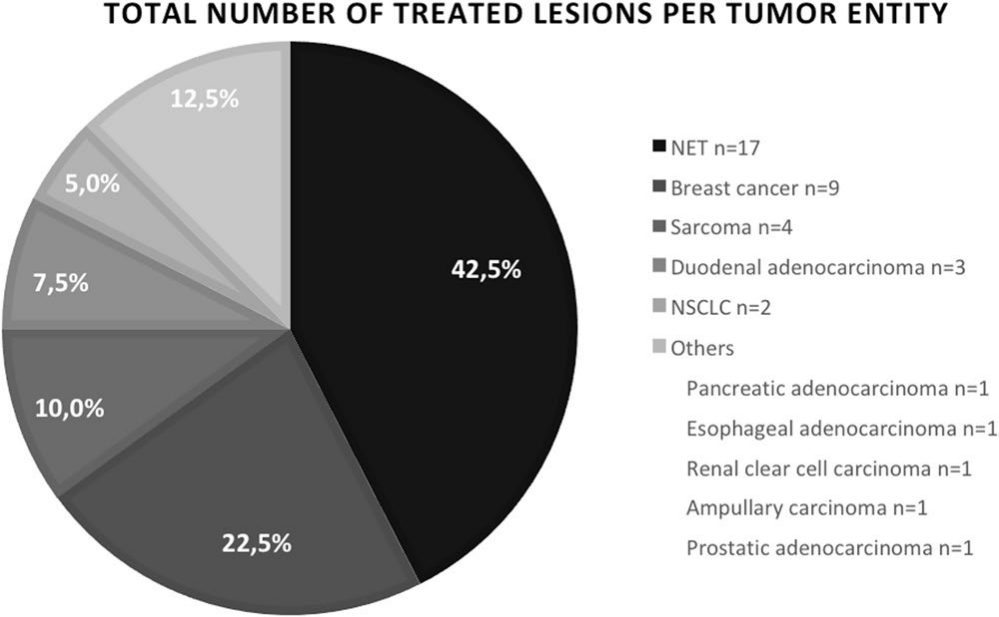
Origin and number of treated liver metastases. *NET* Neuroendocrine Tumor, *NSCLC* Non-small cell lung cancer.

Eight patients had liver metastases originating from a NET, four from breast cancer, three from a sarcoma, two from a non-small cell lung cancer (NSCLC), and one each from a duodenal adenocarcinoma, esophageal adenocarcinoma, pancreatic adenocarcinoma, renal clear cell carcinoma, ampullary carcinoma and prostate carcinoma (Fig. 1).

The decision to use SMWA was always based on the multidisciplinary tumorboard recommendation. A detailed description can be found in Table 2.

**Table 2:** Detailed results per patient.

Patient	Tumor entity	Treatment before SMWA	Tumorboard decision	Systemic treatment at time of SMWA	Follow-up time, months	Number of sessions	Number of treated lesions	Incomplete ablation	Local recurrence	Disease progression in the liver	Disease progression elsewhere
1	NET	Resection of primary tumor, systemic Sandostatin	Ablation by adverse reaction to sandostatin treatment	none	14	1	1				
2	NET	Resection of primary tumor, systemic Sandostatin	Only two lesions, minimal progression under systemic therapy	Sandostatin	17	1	2			X	
3	NET	Resection of primary tumor and Laparoscopic MWA	Single lesion on two separate occasions	none	18	1	1				
4	NET	Resection of primary tumor	Two clearly defined metastasis	none	24	1	2				
5	NET	Resection of primary tumor, systemic Sandostatin	Multiple liver metastasis, ablation of the biggest lesions (debulking)	Sandostatin	19	1	4			X	
6	NET	Resection of primary tumor	Single metastasis in segment I, operative risk too high	none	20	2	4	X		X	
7	NET	Metabolic therapy, resection primary tumor	Multiple liver metastasis, after systemic therapy only one remains	none	22	1	1			X	
8	NET	Resection of primary tumor	To confirm diagnosis, biopsy and ablation of single lesion	none	10	1	1			X	X
9	Breast cancer, Triple-positive	Resection of primary tumor, radiotherapy, hormonal therapy	Single liver metastasis, biopsy indicated by previous rectum carcinoma, ablation in the same session	hormonal therapy	29	1	2			X	X
10	Breast cancer, ER and HER-2 positive	Resection of primary tumor, adjuvant chemotherapy, immunotherapy and radiotherapy	Two liver metastasis, good response under systemic therapy	immunotherapy	15	1	2			X	
11	Breast cancer, HER-2 positive	Resection of primary tumor, adjuvant chemotherapy and immunotherapy	Two vanishing liver metastasis, biopsy and ablation indicated	adjuvant chemotherapy and immunotherapy	14	1	1				
12	Breast cancer, triple-negative	Resection of primary tumor, palliative chemotherapy	Single liver metastasis, young patient. 2^nd^ ablation under chemotherapy, patient wishes	chemotherapy	22	2	4			X	X
13	Sarcoma	Resection of primary tumor, hemihepatectomy and MWA for two lesions in the left liver	No other metastasis, young patient, limited therapeutic options	none	10	1	4			X	
14	Sarcoma	Resection of primary tumor	Ablation for single growing liver metastasis, with stable small pulmonary lesions	none	22	1	1				X
15	Sarcoma	Resection of primary tumor	Single liver lesion, biopsy indicated, concomitant ablation	none	17	1	1			X	X
16	NSCLC	Resection of primary tumor	Single liver lesion, biopsy and concomitant ablation indicated by DD HCC	none	2	1	1				X
17	NSCLC	Resection of primary tumor, adjuvant chemotherapy, Radiosurgery brain metastasis	Long oligometastatic course, systemic therapy contra-indicated, ablation for single lesion	none	7	1	1		X		X
18	Duodenum adenocarcinoma	Resection of primary tumor, adjuvant chemotherapy, palliative chemotherapy for local recurrence	Three isolated liver metastasis two years after end of chemotherapy, young patient	none	10	1	3		X	X	X
19	Esophagus adenocarcinoma	Palliative chemotherapy (liver metastasis diagnosed before primary tumor)	Liver cirrhosis, liver lesion suspicious of HCC, biopsy and concomitant ablation indicated	none	7	1	1		X	X	X
20	Pancreas adenocarcinoma	Palliative chemotherapy and radiotherapy	single lesion, atypical for metastasis from pancreatic adenocarcinoma, biopsy indicated, ablation at the same time	none	7	1	1				
21	Renal clear cell carcinoma	Resection of primary tumor	Single liver lesion, biopsy indicated, and concomitant ablation	none	20	1	1				
22	Prostatic carcinoma	Resection of primary tumor, antiresorbtive and antiandrogen therapy	Single metastasis, origin unclear by previous rectal carcinoma, biopsy and ablation indicated	antiresorbtive and antiandrogen therapy	8	1	1				X
23	Ampullary carcinoma	Resection of primary tumor, adjuvant chemotherapy	Single metastasis, stable months after end of chemotherapy	none	6	1	1		X		X

*MWA* Microwave ablation, *DD* differential diagnosis, *HCC* Hepatocellular carcinoma.

### Intervention data

Most patients underwent one SMWA session; two underwent a second SMWA session, one to treat an incomplete ablation and two new liver lesions, the other to treat a single new liver lesion. In 74% of patients (n = 17), a CT-guided biopsy of the lesion was performed during SMWA, proving the diagnosis in all but two patients, where histology was inconclusive. The smallest successfully biopsied lesion was 7 mm in diameter in this series. One to four lesions were treated per session, with all lesions being smaller than four centimeters in diameter (Table 3). One patient presented with incomplete ablation as diagnosed on the first post-SMWA imaging immediately after the intervention, and confirmed on the follow-up imaging at 3 months. The lesion had to be treated with special care initially, as it was in segment I and close to the vena cava, where the heat sink effect was higher. We successfully treated this incomplete ablation with an irreversible electroporation (IRE) session.

**Table 3:** Results – Intervention.

Variable	
Number of treated lesions, n	40
SMWA sessions, n	25
Median number of session per patient, n (range)	1 (1–2)
Median number of treated lesions per session, n (range)	2 (1–4)
Median lesion size, mm (range)	13.5 (6–39)
Median duration of ablation, minutes (range)	4.75 (1.25–18)
Intraoperative biopsy, n (%)	17 (73.9)
Positive biopsy	15 (88.2)
Incomplete ablation, number of lesions (%)	1 (2.5)
Time to diagnosis of incomplete ablation, months	3
Re-Ablation (IRE), n	1

*IRE* irreversible electroporation.

**Figure 2:**
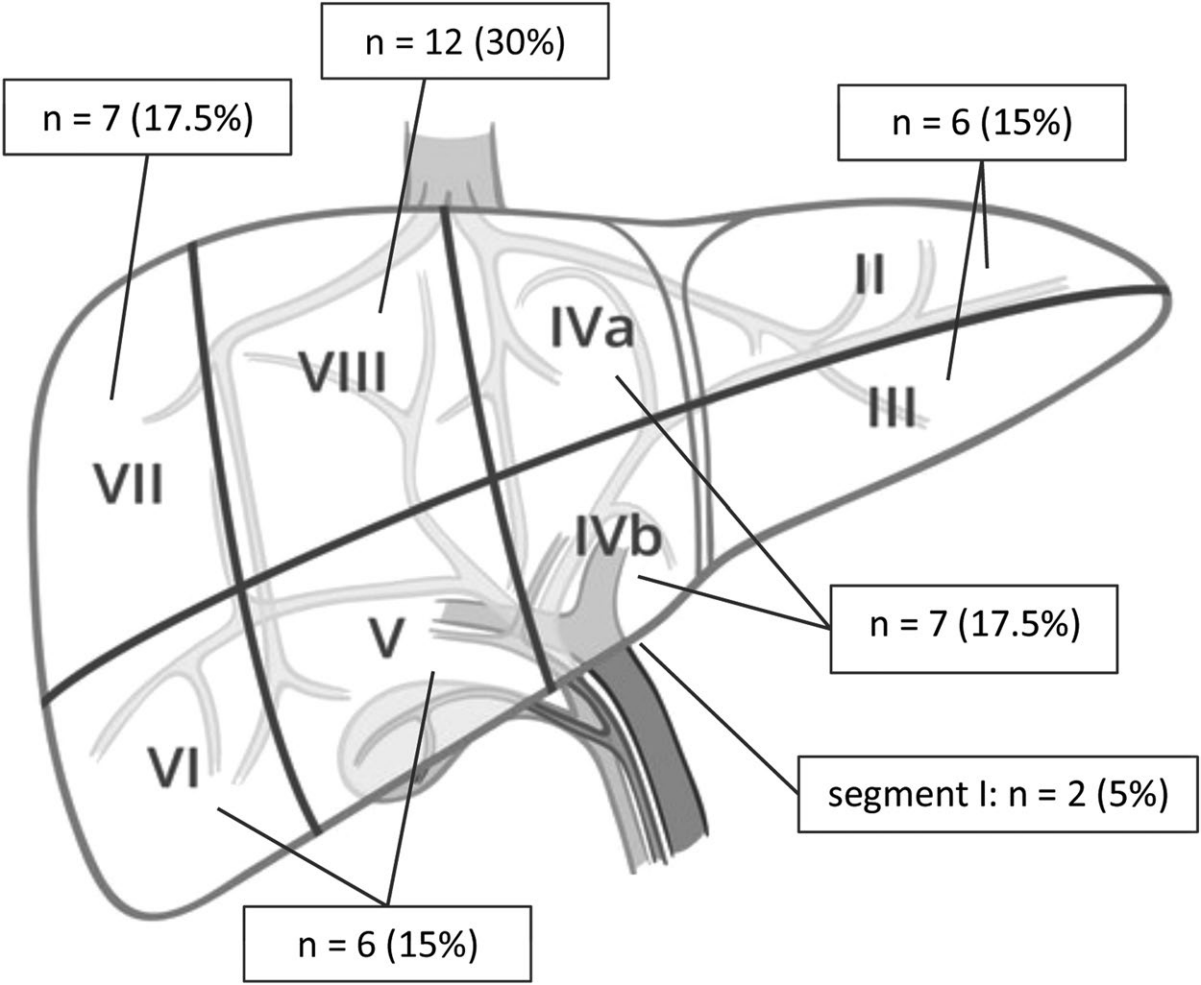
Distribution of treated liver lesions, per segment.

Lesion location did not limit treatment option, with any location within the liver amenable to SMWA. The majority of lesions were located at the liver dome (Segment VII and VIII) and in segment IV, two lesions were treated in segment I (Fig. 2).

### Post-interventional data

Length of hospital stay was two nights for the majority of patients (Table 4). There were no major complications. Three (12%) patients presented with minor complications (≤grade IIIa), including one pleuritis (grade I), one liver abscess requiring rehospitalisation and percutaneous drainage (grade IIIa), and one thrombosis of a branch of the left portal vein necessitating anticoagulation for three months (grade II).

**Table 4:** Post-interventional data.

Variable	
Median LOS, days (range)	2 (2–14)
Number of patients with complications, n (%)	3 (12)
Dindo-Clavien classification	
Grade I	1
Grade II	1
Grad IIIa	1
Median duration of follow-up, months (range)	15 (2–32)
Local recurrence at ablation site, number of lesions (%)	4 (10)
Time to diagnosis, months	3
Re-Ablation, n	0
Overall disease progression, n (%)	17 (74)
Intrahepatic only, n (%)	5 (29)
Extrahepatic only, n (%)	4 (24)
Intra- and Extrahepatic, n (%)	8 (47)
Deceased during follow-up, n (%)	7 (30)
Disease-free survival, median in months (range)	7 (0–26)
Overall survival, median in months (range)	18 (2–39)

*LOS* Length of stay.

Median follow-up was 15 months (2–32). We observed local recurrence in four patients, diagnosed on the first follow-up scan at three months. All four patients also presented with disease progression – intra- and/or extrahepatic - prompting a change in therapeutic strategy, so that no re-ablation was attempted. Overall disease progression was observed in 74% of patients (n = 17), at a median of seven months after SMWA (range 0–25). Isolated intrahepatic disease progression occurred in five patients.

Seven patients died of disease progression during the follow-up period, between 2 and 25 months after SMWA. The patient who died two months after SMWA had NSCLC, and cerebral metastases were diagnosed shortly after the intervention.

## Discussion

In this study, we are able to demonstrate that stereotactic ablation is a very safe and technically feasible treatment for patients with malignant liver lesions. Using a navigated, percutaneous approach not only allows for the treatment of lesions otherwise not amenable to ablation (such as lesions located close to major vessels and bile ducts, subcapsular, subdiaphragmatic liver dome lesions, or lesion located in segment I). It also allows for simultaneous, diagnostic biopsy of very small (sub-centimetric) tumors within the liver. Being able to precisely biopsy and simultaneously ablate very small lesions is of particular interest in a patient population with a history of previous cancer or in the setting of concomitant tumors, where determining the origin of the liver metastases at a very early stage is a prerequisite for correct and rapid diagnosis and treatment. This concurs with currently available literature reports, which show that the use of a navigation system improves precision of needle placement and reduces multiple needle repositioning^12–14^.

SMWA can be safely performed in patients while under systemic therapy, without additional risks and without the need to stop the therapy. In this setting, SMWA may be seen as an adjunct to a systemic therapy or other local treatment strategies.

In contrast to more invasive local treatment options such as surgical tumor resection, SMWA is tolerated very well, with the vast majority of patients not suffering from any postoperative symptoms or complications, and a short hospital-stay. Postinterventional intrahepatic thrombosis have been reported in patients treated by thermal ablation close to a blood vessel^19–21^, and portal veins are particularly susceptible to thrombosis^21^, which could have serious consequences in patients with little hepatic reserve, due to underlying liver disease or previous interventions. Following this complication, we elected to treat all patients with prophylactic anticoagulation if the ablation had been performed close to a major blood vessel. One patient with a hepaticojejunostomy after a pancreaticoduodenectomy presented with an abscess in the ablation zone and was treated with antibiotics. This population has been identified as at higher risk for post-ablation abscesses^22,23^. Following this, we implemented a prophylactic antibiotic regimen in all patients undergoing SMWA. No further abscesses were seen. Further studies are needed to determine if this is necessary, or if prophylactic antibiotics should be reserved for high-risk patients. Other common complications include injury to the biliary tract, pleural effusion and liver dysfunction, which did not occur in this series^24,25^. Whether or not this can be attributed to the use of navigation technology needs to be further analyzed.

Cancer treatment is becoming increasingly personalized. In selected patients with oligometastatic disease in whom the classical systemic therapies fail to offer complete tumor response, SMWA may be an alternative treatment for local tumor control of single active tumor lesions. SMWA also allows for simultaneous acquisition of tissue for histopathological and molecular analyses, in particular in the context of now increasingly available tumor-targeted treatments^26^.

However, in our patient cohort, ultimately 3/4 of patients presented with some form of disease progression in the shorter or longer term. This stresses the importance of patient selection, so that the indication for SMWA or any other local treatment for hepatic metastases has to be made in the setting of a multidisciplinary tumorboard. Whether or not SMWA results in prolonged patient survival in the setting of stage IV cancer cannot be answered within this cohort.

Particularly in the setting of symptomatic hepatic NET metastases, SMWA offers a low-risk, tissue-sparing treatment option that can be repeated in case of tumor recurrence. At the moment, treatment with curative intent consists in liver resection, and has shown to improve survival^9,10^. Palliative therapeutic strategies for symptomatic disease also include surgical resection, sometimes as a debulking procedure. However, given the tendency for recurrence, a safe, minimally invasive solution is needed. Available data shows that RFA of NET liver metastases may be beneficial, but to this day, there is little evidence on the efficacy of SMWA of these lesions^11^. SMWA was performed in our patient series with stable disease, in symptomatic patients where systemic therapy was contra-indicated or when biopsy of a single lesion was needed for diagnostic purposes.

As for other, non-CRLM and non-NET liver metastasis, the benefit of resection remains unproven and there is currently few data on the oncological benefit of a local therapy for such patients^10,27–32^. Surgical resection might be beneficial in selected patients with well-controlled metastatic disease under systemic therapy, but with a significant morbidity^27^. In breast cancer for example, repeated histology becomes increasingly important for targeted therapy^33^. In this situation, adding local treatment to navigated biopsy of a liver metastasis is an interesting option.

## Conclusion

Percutaneous SMWA is a safe and technically feasible treatment for NCRLM, which can be repeatedly performed in a low-risk minimally invasive setting and as an adjunct to systemic therapy, particularly if the lesion is unresectable or conventionally unablatable. SIMWA combines a precise biopsy (diagnostics), even for small, sub-centimetric lesions, with the actual ablation (treatment). Even in the increasingly important era of personalized and patient-oriented medicine, the indication for SMWA must always be determined by a multidisciplinary tumorboard.

Clearly, further studies are needed to validate these results and to analyze the long-term oncological and quality of life benefit for these patients.
